# A Novel Spontaneous Mutation of the *SOX10* Gene Associated with Waardenburg Syndrome Type II

**DOI:** 10.1155/2020/9260807

**Published:** 2020-08-28

**Authors:** Sen Chen, Yuan Jin, Le Xie, Wen Xie, Kai Xu, Yue Qiu, Xue Bai, Hui-Min Zhang, Xiao-Zhou Liu, Xiao-Hui Wang, Wei-Jia Kong, Yu Sun

**Affiliations:** ^1^Department of Otorhinolaryngology, Union Hospital, Tongji Medical College, Huazhong University of Science and Technology, Wuhan 430022, China; ^2^Institute of Otorhinolaryngology, Tongji Medical College, Huazhong University of Science and Technology, 430022 Wuhan, China

## Abstract

Waardenburg syndrome (WS), also known as auditory-pigmentary syndrome, is the most common cause of syndromic hearing loss. It is responsible for 2–5% of congenital deafness. WS is classified into four types depending on the clinical phenotypes. Currently, pathogenic mutation of *PAX3*, *MITF*, *EDNRB*, *EDN3*, *SNAI2*, or *SOX10* can cause corresponding types of WS. Among them, *SOX10* mutation is responsible for approximately 15% of type II WS or 50% of type IV WS. We report the case of a proband in a Chinese family who was diagnosed with WS type II. Whole exome sequencing (WES) of the proband detected a novel heterozygous spontaneous mutation: *SOX10* c.246delC. According to analysis based on nucleic acid and amino acid sequences, this mutation may produce a truncated protein, with loss of the HMG structure domain. Therefore, this truncated protein may fail to activate the expression of the *MITF* gene, which regulates melanocytic development and plays a key role in WS. Our finding expands the database of *SOX10* mutations associated with WS and provides more information regarding the molecular mechanism of WS.

## 1. Introduction

Waardenburg syndrome (WS), also known as auditory-pigmentary syndrome, is one of the most common causes of syndromic deafness, associated with 2–5% of congenital deafness cases [1]. According to correlational research, sporadic cases of WS have been reported worldwide; the highest reported incidence is among Kenyan Africans, who have an incidence of 1 in 20,000 [2]. WS accounts for approximately 1% of the deaf population in China. Basic clinical symptoms of WS include dystopia of the canthus; abnormal pigmentation of the skin, hair, and eyes; different degrees of unilateral or bilateral sensorineural deafness; and a high and wide nasal base. The disease is divided into four types; type I exhibits only these basic symptoms. The clinical manifestations of type II are basically the same as type I, but without dystopia of the canthus. The clinical manifestations of type III are the same as type I, but combined with upper limb deformity, while type IV exhibits basically the same symptoms as type II, but combined with Hirschsprung disease (gastrointestinal malformation) [3–6]. Mutations of the *PAX3*, *SOX10*, *MITF*, *SNAI2*, *EDNRB*, and *EDN3* genes have been found in cases of the different types of WS [7, 8]. According to previous reports, *SOX10* mutations cause approximately 15% of type II WS and 45–55% of type IV WS [9, 10]. To date, the total number of reported *SOX10* pathogenic mutations related to WS is 82, according to the Leiden Open Variation Database (LOVD) (https://grenada.lumc.nl/LOVD2/WS/home).

The *SOX10* gene (SRY- (sex-determining region Y-) box 10) is located on chromosome 22q13.1. It contains five exons and encodes a protein which consists of 466 amino acids, with a relative molecular weight of approximately 51,000 [11]. *SOX10* belongs to the *SOX* (SRY-related HMG-box) family, and it is first expressed in the dorsal neural tube at the early stage of neural crest cell (NCC) migration. With differentiation of the NCC, *SOX10* begins to be widely expressed throughout the adult body, such as in the hair follicles, inner ear, iris, and gastrointestinal tract. *SOX10* acts as a transcription factor of the microphthalmia-associated transcription factor (*MITF*) gene, and MITF plays a key role in the development of melanocytes [12]. Currently, it is believed that the aetiology of WS is caused by the abnormal development of NCC [7, 13, 14]. Additionally, the hearing loss of WS may relate to the abnormal proliferation, survival, differentiation, or migration of NCC-derived melanocytes (a type of intermedia cells of the stria vascularis) [15]. In the mouse inner ear, the *SOX10* gene starts to express in the otic vesicle of the cochlea at embryonic day 9.5-12.5 (E9.5-E12.5), and the expression is restricted to supporting cells (SCs) of the cochlear epithelium, glial cells, and marginal and intermedia layers of the stria vascularis after birth [16–18]. Knockout of the *SOX10* gene leads to the death of NCC-derived Schwann cells in a mouse model [19]. However, the role that it played in supporting cells is still unknown.

As WS has a lot of genetic heterogeneity, the molecular mechanism of WS needs better understanding, and more cases of gene mutation associated with WS need to be collected. Here, we report a novel heterozygous *SOX10* mutation in a Chinese family, which provides more information about the molecular diagnosis of WS.

## 2. Materials and Methods

### 2.1. Family Description

**Figure 1:**
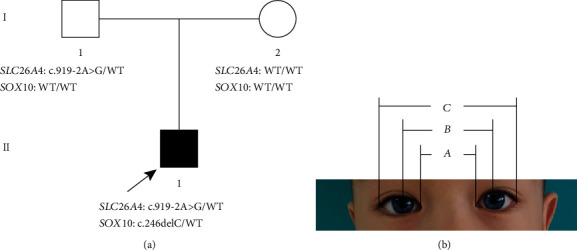
Family pedigree and clinical features of the proband. The (a) pedigree indicates that Family II-1 had a spontaneous heterozygous mutation (*SOX*10 c.246delC), which is marked black. Family I-1 only carried the mutation of the SLC26A4 gene. (b) The iris heterochromia in both eyes of the proband, which are blue. *a*, *b*, and *c* indicate the distance between the inner canthi, pupils, and outer canthi. WT: wild type.

Family member II-1: a seven-year-old boy, who failed to pass hearing screening and who had white hair at the front of his forehead and bilateral blue irises, was diagnosed with type II WS. Neither parent of the boy exhibited similar symptoms (Figure 1(a) and Table 1).

### 2.2. Clinical Examination

The proband underwent audiological examination including otoscopic examination, pure tone audiometry, auditory brainstem response (ABR), distortion product otoacoustic emissions (DPOAE), and auditory immittance and auditory steady-state evoked responses (ASSR). A computed tomography (CT) scan of the proband was also performed. The distance between the inner canthi (*a*) of the p5roband was measured at 29 mm, the distance between the pupils (*b*) was 54 mm, and the distance between the outer canthi (*c*) was 89 mm. The *W* exponent was obtained according to the formulae *X* = (2*a* − 0.2119*c* − 3.909)/*c*; *Y* = (2*a* − 0.2479*b* − 3.909)/*b*; and *W* = *X* + *Y* + *a*/*b* (Figure 1(b)) [20].

### 2.3. Mutation Detection and Analysis

Targeted sequencing and Sanger sequencing were performed by BGI Genomics (Wuhan, China). After obtaining informed consent, we collected 3–5 mL of venous peripheral blood from the proband and his parents to prepare DNA. Genomic DNA from all the family members was extracted according to the manufacturer's standard procedure using the QIAamp DNA Blood Midi Kit (Qiagen Inc., Hilden, Germany). The genomic DNA of the family was fragmented using a Covaris LE220 ultrasonicator (Covaris Inc., Woburn, Massachusetts, USA) to generate a paired-end library (200–250 bp). Following array hybridization, elution, and postcapture amplification, the library was enriched. An Agilent 2100 Bioanalyzer and ABI StepOne were used to estimate the magnitude enrichment of the products. Subsequently, the amplified libraries were used for circularization and sequencing on the BGISEQ-500 platform. For circularization, PCR products with different barcodes were pooled together at equimolar concentrations to yield a final amount of 80 ng. Each pool was subsequently heat-denatured, and the single-strand DNA was mixed with an MGIEasy™ DNA Library Prep Kit V1 (PN: 85–05533-00, BGI, Shenzhen, China) to form a 60 *μ*L reaction system, which was subsequently incubated at 37°C for 30 minutes. Finally, 20 *μ*L of each single-circle-library pool was used as input to prepare the DNB. Each pool was then sequenced on one lane, using 100SR chemistry with a BGISEQ-500RS high-throughput sequencing kit (PN: 85–05238-01, BGI). After sequencing, the data were automatically demultiplexed by index. The “clean reads” (with a length of 90 bp) derived from targeted sequencing and filtering were then aligned to the human genome reference (hg19) using the BWA (Burrows Wheeler Aligner) Multi-Vision software package. After alignment, the output files were used to perform sequencing coverage and depth analysis of the target region, single-nucleotide variants (SNVs), and INDEL calling. We detected SNVs and indels using GATK software. All SNVs and indels were referenced and compared with multiple databases, including the National Center for Biotechnology Information (NCBI) GenBank database (https://www.ncbi.nlm.nih.gov/nuccore/), the Database of Single Nucleotide Polymorphisms (dbSNP) (http://www.ncbi.nlm.nih.gov/projects/SNP/), and the 1000 Genomes Database (https://www.internationalgenome.org). According to the high-throughput sequencing results, Sanger sequencing was performed to confirm whether his parents had the same mutations.

## 3. Results

### 3.1. Clinical Data

**Figure 2:**
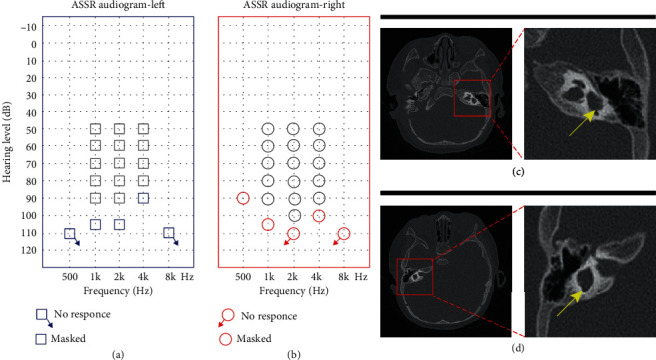
Clinical audiology examination and temporal bone CT scan of the proband. (a) ASSR (auditory steady-state responses) of the left ear: 105 dB, 105 dB, and 90 dB at 1, 2, and 4 kHz, respectively; the remaining frequencies showed no response. (b) ASSR of the right ear: 90 dB, 105 dB, and 100 dB at 500 Hz, 1, and 4 kHz; the remaining frequencies showed no response. (c) Semicircular canal abnormalities shown on high-resolution axial CT in the red square (left ear). (d) Semicircular canal abnormalities shown on high-resolution axial CT in the red square (right ear).

The proband had white hair at the front of his forehead and bilateral blue irises. His parents had no pigmentary abnormalities in the skin, the hair, and the eye or any other WS-associated phenotypes. The audiology examination of the proband showed failed bilateral otoacoustic emissions; all bilateral ABR thresholds were over 105 dB nHL; ASSR showed the thresholds of the left ear were 105 dB nHL at 1 kHz, 105 dB nHL at 2 kHz, and 90 dB nHL at 4 kHz, while the thresholds of the right ear were 90 dB nHL at 500 Hz, 105 dB nHL at 1 kHz, and 100 dB nHL at 4 kHz (Figures 2(a) and 2(b)). The temporal bone CT scan suggested that the shape and size of the bilateral cochleae were not obviously abnormal; however, the middle and top circles of the cochleae were obscurely decomposed, the vestibule was slightly enlarged on both sides, all the right semicircular canals were fused with the vestibule, the left posterior and superior semicircular canals were short, and the horizontal semicircular canal was fused with the vestibule (Figures 2(c) and 2(d)). We substituted the measured distances of inner canthi (*a*), pupil (*b*), and outer canthi (*c*) into the *W* exponent formula (as introduced in Section 2.2) and obtained that the *W* exponent was 1.687, less than 1.95 (Figure 1(b)).

### 3.2. Mutation Identification Data

**Figure 3:**
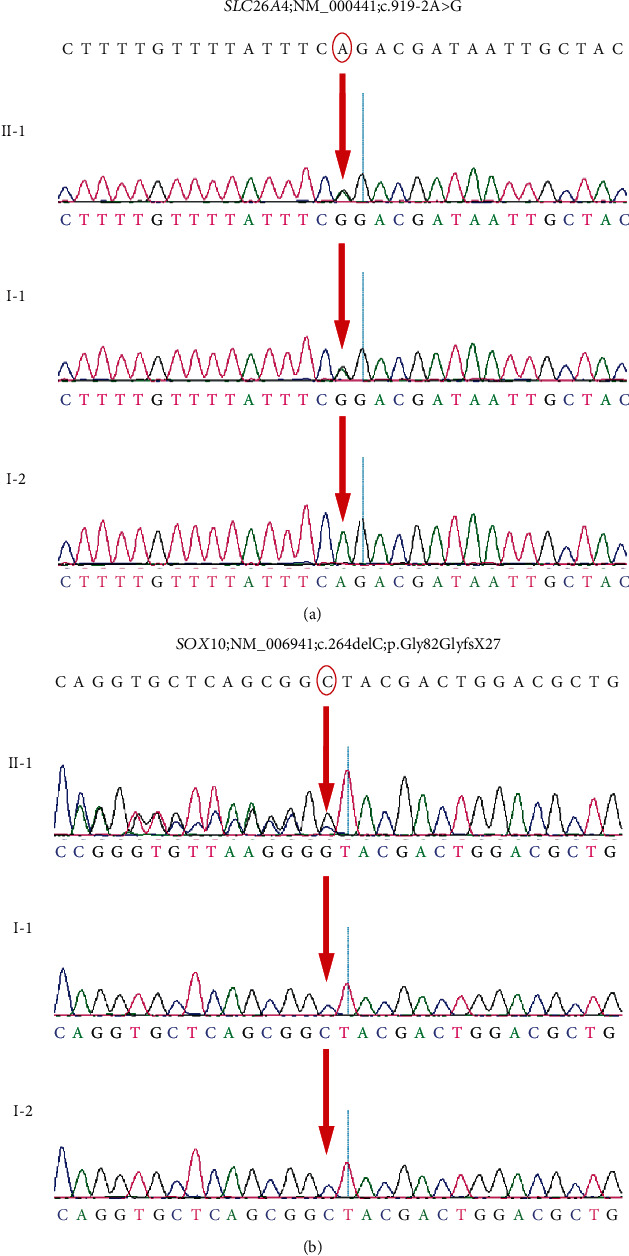
Genetic sequencing results of the proband and his parents. The red arrow indicates the site of the base deletion or substitution.

The NGS results were compared with the human reference genome (GRCh37/hg19). The proband carried two heterozygous mutations: the *SOX10* c.246delC and the *SLC26A4* c.919-2A>G; the c.246delC mutation was a truncation with deletion of the no. 246 nucleotide cytosine, occurring in EX2/CDS1 of *SOX10*. No report related to the c.246delC mutation in *SOX10* was found in the HGMD (Figure 3(b)). The c.919-2A>G mutation was a splice mutation of no. 919-2 nucleotide from adenine to guanine, occurring in intron7 of *SLC26A4* (Figure 3(a)). The father of the proband only carried the mutation of the *SLC26A4* gene, at the same mutation site as that of the proband, while the mother of the proband had the wild-type *SOX10* and *SLC26A4* genes.

### 3.3. Functional Analysis of the Mutant Protein

**Figure 4:**
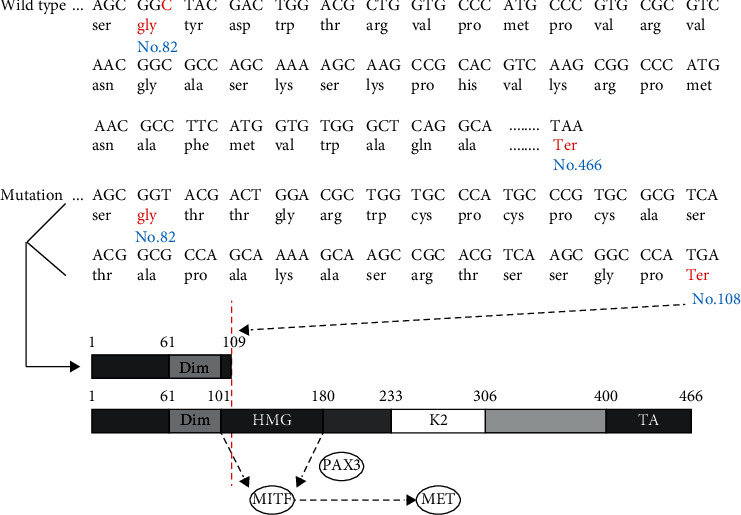
Amino acid coding diagram for the proband and schematic diagram of the *SOX10* gene domain. The red letters indicate the changed amino acids and the site of the stop codon. The mutation caused early termination of the coding sequence. If a deletion mutation occurs in front of the HMG domain, the HMG domain cannot combine with the promoter of the target gene (*MITF*).

The *SOX10* gene contains three main functional domains: a SOX Group E domain which is highly conserved, the carboxy terminal (C-terminal) transactivation (TA) domain, and a highly conserved and highly active component domain: the highly mobility group (HMG) (102–181 amino acids). The mutation identified in the proband occurred in amino acid no. 82, with deletion of nucleotide C in position no. 246 causing a frameshift mutation. Consequently, the original glycine encoded by GGC at nucleotide nos. 244–246 was changed into glycine encoded by GGG. Following the change at no. 82 glycine, the no. 108 amino acid was converted into a termination codon (Figure 4).

## 4. Discussion

A diagnosis of type II WS was established in the proband. In terms of clinical diagnosis, audiology testing showed that the proband suffered from profound bilateral congenital sensorineural deafness; we calculated a *W* index of 1.687, less than 1.95, indicating that the proband had no dystopia of the canthus; the proband had white hair on his head and had bilateral blue irises at birth, but no digestive tract abnormalities [21]. In terms of the genetic test results, we found two genetic mutations in the proband; the S*LC26A4* gene related to the syndrome type deafness with vestibular pipe expansion showed autosomal recessive inheritance. According to a previous report, in the appropriate clinical context, bilateral agenesis or hypoplasia of the semicircular canals or both, associated with an enlarged vestibule and cochlear deformity, strongly suggests a diagnosis of WS linked to a *SOX10* mutation, so we speculate that the spontaneous *SOX10* c.246delC mutation may be the cause of the type II WS of the proband [9]. Previous studies have suggested that type II WS patients with *SOX10* mutations have a very high spontaneous mutant rate [22].

Sensorineural hearing loss is a common clinical phenotype in WS patients with *SOX10* mutations. In the inner ear, most of the hearing loss induced by gene mutations, ototoxic drugs, and aging is caused by the hair cell malfunction [23–27]. A previous study showed that *SOX10* mutation will cause both hair cell and SC loss in a heterozygous Dom mouse model [15]. In pigs, shorter cochlear conduct was induced by *SOX10* p.Arg109Trp missense mutation [28]. There are complex regulatory networks between SCs and hair cells [29, 30]. Although *SOX10* is only expressed in SCs, it may affect the survival of hair cells by regulating the function of SCs. Therefore, more detailed observations should be made to explore the effect of the *SOX10* gene on SC function. Moreover, SC-targeted gene therapy can be tried in a murine model [31]. *SOX10* is a key transcription factor in the migration and differentiation of NCC, and its mutations lead to abnormal differentiation of NCC-derived melanocytes, which results in abnormal pigment distribution and deafness and is the main cause of WS [32]. *SOX10* can exert its function by binding to the promoter or enhancer of the target gene alone or together with other transcription factors. *MITF*, *TYR*, *TYRP1*, *DCT*, *MPZ*, *GJB1*, *RET*, *DCT*, and *EDNRB* are the downstream target genes directly regulated by *SOX10*. These target genes are directly or indirectly involved in melanin synthesis, among which *MITF* is a key regulatory gene for melanocyte development and melanin synthesis. *SOX10* can act alone or directly with *PAX3* to generate a coeffect to stimulate and upregulate the expression of *MITF* [12, 33]. The *SOX10* c.246delC mutation resulted in early termination of the coding protein sequence at amino acid position 108, and consequently, the mutant protein did not contain the HMG domain and the TA domain. The main function of the HMG domain is to identify and bind the promoter of the target gene [34] (Figure 4), so the heterozygous *SOX10* c.246delC mutant protein could not effectively activate the *MITF* promoter, causing a decrease in effective MITF protein expression, leading to an insufficient dose effect, and resulting in disordered melanocyte development and abnormal melanin synthesis. The main function of melanocytes is to produce melanin to ensure the pigmentation of hair and skin. Melanocytes developed from NCC are widely expressed in the dermis, epidermis, vascular striae of the inner ear, and choroid of the eye, and their developmental disorder will lead to WS characterized by hearing loss and abnormal distribution of pigment in the skin and hair [12, 35]. The mutation *SOX10* c.246delC (exon 2 in NM_006941) has not been reported according to the Human Gene Mutation Database (HGMD) (http://www.hgmd.cf.ac.uk/ac/index.php) [7].

In addition, through analysis of the reported cases and the literature, we found that when the *SOX10* mutation site occurred behind the 180th amino acid, or you could say after the HMG domain, it caused more severe symptoms of type IV WS [5, 6, 36–39] (Table 2). We speculate that the reason may be due to the truncated mutation which means that the HMG domain loses the normal function of the protein, but can combine with the target gene promoter. It then competes with the normal protein for binding sites and restrains the influence of the normal protein, leading to more serious consequences. The exact molecular mechanism remains to be confirmed.

## 5. Conclusion

We identified a new mutation site in the *SOX10* gene, explored the possible pathological mechanism of the clinical phenotype caused by this mutation, expanded the database of WS pathogenic gene mutations, and deepened the association between the mutation site and the clinical phenotype, so as to further explore the molecular pathogenic mechanism of WS.

## Figures and Tables

**Table 1:** Genetic variants found in this family.

Gene	Variant	Protein level	Type	Father	Mother	Proband
*SOX10*	c.246delC	p.Giy82GlyfsX27	Heterozygous	Normal	Normal	Heterozygous
*SLC26A4*	c.919-2A>G	Splicing	Heterozygous	Heterozygous	Normal	Heterozygous

**Table 2:** The mutation of *SOX10* in WS4 probands in the literature.

Gene	Nucleotide changes	Amino acid changes	Exon	WS subtype	Reference
*SOX10*	c.1333deIT	p.Ser445Glnfs∗57	5	WS4	6
*SOX10*	c.1107ins19	p.Thr370Serfs∗38	5	WS4	40
*SOX10*	c.752_753ins7	p.Gly252Alafs∗31	5	WS4	41
*SOX10*	c.895delC	p.Gln299Serfs∗12	5	WS4	5

## Data Availability

The data used to support the findings of this study are included within the article.
